# Beneficial Changes in Apolipoprotein Concentrations After Bariatric Surgery in Obese Women

**DOI:** 10.3390/metabo16070496

**Published:** 2026-07-14

**Authors:** Bartłomiej Łukaszuk, Adrian Chabowski, Andrzej Ziemba, Barbara Choromańska, Piotr Myśliwiec, Katarzyna Supruniuk, Agnieszka Mikłosz

**Affiliations:** 1 Department of Physiology, Medical University of Bialystok, 15-222 Bialystok, Poland; adrian.chabowski@umb.edu.pl (A.C.); agnieszka.miklosz@umb.edu.pl (A.M.); 2 Clinical and Research Department of Applied Physiology, Mossakowski Medical Research Centre, Polish Academy of Science, 02-106 Warsaw, Poland; ziemba@imdik.pan.pl; 3 1st Clinical Department of General and Endocrine Surgery, Medical University of Bialystok, 15-276 Bialystok, Poland; choromanska.barbara@gmail.com; 4 Department of Minimally Invasive Surgery, Medical University of Bialystok, 15-276 Bialystok, Poland; piotr.a.mysliwiec@gmail.com; 5 Department of Biology and Medical Genetics, Medical University of Gdańsk, 80-210 Gdańsk, Poland; katarzyna.supruniuk@gumed.edu.pl

**Keywords:** apolipoproteins, obesity, metabolic syndrome, bariatric surgery, ApoB/ApoA1

## Abstract

Background: Lipoproteins are molecules composed of phospholipids and apolipoproteins that transport triacylglycerol and cholesterol in blood and are implicated in the development of many diseases. Methods: In this study, we fill a knowledge gap by precisely characterizing the apolipoprotein profile (with Bio-Plex Human Apolipoprotein Assay) in three metabolically separate groups of individuals (lean individuals and obese individuals without and with metabolic syndrome) and at four distinct time points (0, 3, 6, and 12 months post bariatric surgery). Results: Obese patients had a higher baseline ApoB/ApoA1 ratio, which returned to the reference level over the follow-up period. The above is of clinical importance, as the ratio is a predictor of adverse cardiovascular events common in obese subjects. Interestingly, plasma concentrations of most of the investigated apolipoproteins appeared to be relatively stable at the onset of the experiment, with changes observed later in time. We detected significant drops in the levels of ApoC3, ApoD, and ApoH that occurred as early as three months post intervention. On the other hand, the levels of ApoA2, ApoE, and ApoJ increased with time. Of the above, ApoE is known to be involved in the removal of TAGs and cholesterol from the blood. Conversely, ApoJ appears to be one of the determinants of the tissues’ responsiveness to insulin. Thus, it may be an indicator of the reduced insulin resistance observed a few months after the surgery. Conclusions: Overall, the investigated proteins have a documented role in the development and progression of vascular pathologies. Hence, our results may be interpreted as a sign of improved cardiovascular fitness of our patients.

## 1. Introduction

Triacylglycerols (TAGs) and cholesterol are essential lipids in the human body. They serve as energy providers (TAGs) and building blocks for cell membranes and hormones (cholesterol) [1,2].

The functionality of lipoproteins is strongly dependent on their apolipoprotein composition. For instance, Nissen et al. [3] tested ApoA-1 and its impact on atheroma (atherosclerotic plaque) formation. Specifically, the researchers investigated ApoA-1 Milano, a natural variant of the protein present in an Italian village whose inhabitants are characterized by longevity and a relatively low degree of atherosclerosis, both despite the very low HDL-C levels in their blood [3]. The authors demonstrated that a 5-week infusion of ApoA-1 Milano complexes led to a decrease in atheroma volume, with no such changes reported for the placebo group (double-blind design). This observation is also supported by a study by Geh et al. [4], which indicated that ApoA1 may be dissociated from HDL particles and bind to LDL. Such a translocation reduces the ability of the latter to bind with proteoglycans (a key step in the development of atherosclerosis).

Apolipoproteins act as essential structural components and functional “biochemical keys” for lipoproteins, directly influencing lipid metabolism, receptor recognition, and enzymatic regulation. They can be involved in the generation of the obese phenotype, acting as markers for metabolic syndrome and the differentiation between metabolically healthy obese (MHO) and metabolically abnormal obese (MAO) individuals. In line with that notion, Wang et al. [5] found that the ApoB/ApoA1 ratio can be used as a superior marker for evaluating cardiovascular risk in obese individuals, as it directly reflects the balance between atherogenic (ApoB-containing) and anti-atherogenic (ApoA1) apolipoproteins. Besides ApoB, ApoC-III is also a hallmark of atherogenic dyslipidemia associated with obesity and metabolic syndrome. ApoA-I, in turn, acts as a protective factor against obesity, promoting energy expenditure by enhancing lipolysis in white adipose tissue and increasing the expression of uncoupling protein 1 (UCP1) in brown adipose tissue [6]. Herein, we use a longitudinal study to evaluate the concentration of nine apolipoproteins (ApoA1, ApoA2, ApoB, ApoC1, ApoC3, ApoD, ApoE, ApoH, and ApoJ) in lean and obese postmenopausal women (with and without the accompanying metabolic syndrome). All the assessments were conducted at four different time points (0, 3, 6, and 12 months) after bariatric surgery and superimposed on the patients’ glycemic and lipid profiles.

## 2. Materials and Methods

### 2.1. Patients

The study included 52 morbidly obese postmenopausal women (mean age: 51.7 years) with class 3 obesity (BMI > 40 kg/m^2^) who underwent elective bariatric surgery—laparoscopic sleeve gastrectomy at the 1st Department of General and Endocrine Surgery at the University Clinical Hospital in Białystok. The hospital is located in northeastern Poland. All the participants in this study were of Polish origin (West Slavic ethnic group). Morbidly obese patients were divided into two subgroups: morbidly obese without metabolic syndrome (*n* = 20; group designation: Obese MetSx-) and morbidly obese patients with metabolic syndrome (*n* = 32; group designation: Obese MetSx+). The blood samples were collected before surgery (0 m) and 3 months (3 m), 6 months (6 m), and 12 months (12 m) after the bariatric treatment. A diagnosis of metabolic syndrome [7] requires at least three of the following five characteristics: (1) waistline > 89 cm, (2) blood triacylglycerol concentration ≥ 150 mg/dL, (3) blood HDL cholesterol concentration < 50 mg/dL, (4) blood pressure ≥ 130/85 mmHg, and (5) fasting blood glucose concentration ≥ 100 mg/dL. Among obese patients, *n* = 36 women suffered from hypertension, whereas *n* = 52 had insulin resistance (HOMA-IR > 2.0, Table 1). Morbidly obese patients suffering from hypertension received the following medications: ACE inhibitors (lisinopril and perindopril), diuretics (indapamide), calcium channel blockers (amlodipine), and β blockers (bisoprolol and metoprolol). Patients with T2DM received insulin, metformin, and/or gliclazide.

The lean group (*n* = 20; designation of the group: Lean) consisted of healthy postmenopausal women (mean age: 50.9 years; BMI < 25 kg/m^2^) scheduled for elective laparoscopic cholecystectomy. The group included patients with clear medical evidence of gallbladder disease (symptomatic cholelithiasis). The patients in this group were lean and had a chronic form of gallbladder disease without the accompanying conditions (kidney or endocrinological diseases). The patients gave their informed consent to participate in the study. The lean group was of an age similar to that of the patients from the study group (*p* > 0.05, Table 1).

**Figure 1:**
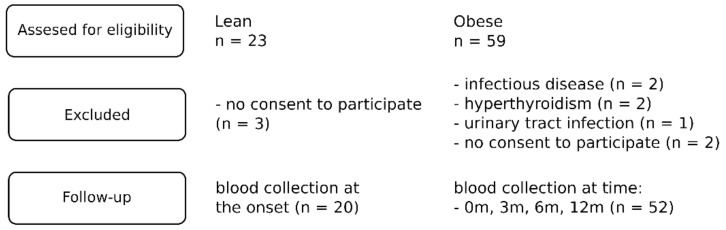
Flow chart of the study.

The study was conducted in accordance with Good Clinical Practice Guidelines and the Declaration of Helsinki [8] and approved by the Ethics Committee of the Medical University of Bialystok (permission no. R-I-002/187/2017). All participants provided informed consent to participate in the study. Eligible participants underwent clinical examination, anthropometric measurements, and appropriate laboratory tests (Table 1). Lean and morbidly obese patients with a positive medical history of acute inflammatory diseases and malignancy were excluded from the study. The participants were non-smokers and did not drink alcohol more frequently than once a month. Exclusion criteria for both lean and morbidly obese women were as follows: malignancy; acute inflammatory diseases; infectious diseases (e.g., HIV/AIDS; hepatitis A, B, or C); autoimmune diseases (Crohn’s and Hashimoto’s diseases, ulcerative colitis); cardiovascular diseases (except for arterial hypertension in the Obese MetSx+ group); digestive-, respiratory-, or genitourinary-system diseases. Ten patients across both groups (7 in the obese group and 3 in the lean group) were excluded based on the study’s criteria (Figure 1).

### 2.2. Blood Collection

Blood samples from obese patients were collected before bariatric surgery, and blood samples from lean patients were collected before laparoscopic cholecystectomy in the overnight fasting state, collected in ethylenediaminetetraacetic acid (EDTA)-coated tubes and serum collection tubes (S-Monovette SARSTEDT). Samples were centrifuged for 10 min at 4 °C and 4000 rpm. The serum and plasma samples were stored at −80 °C until final analysis.

### 2.3. Determination of the Apolipoprotein Concentrations

Apolipoprotein concentrations (Apo-A1, Apo-A2, Apo-B, Apo-C1, Apo-C3, Apo-D, Apo-E, Apo-H, and Apo-J) were quantified using the Bio-Plex Pro Human Apolipoprotein 10-Plex Assay (Bio-Rad Laboratories, Inc.; Hercules, CA, USA) according to the manufacturer’s instructions. Briefly, the serum samples were centrifuged at 1000 rpm for 15 min at 4 °C to clear the samples of precipitate, then immediately diluted (1: 50,000) with sample dilution buffer. Thirty µL of standards, controls, blanks, and samples was added to the appropriate microplate well. Then, 10 µL of capture beads was added to each well, and the plate was incubated on a shaker at 850 ± 50 rpm for 1 h at room temperature. After incubation, the plate was washed three times with diluted assay buffer. Reconstituted detection antibodies (40 µL) were added to each well, and the assay plate was incubated a second time for 1 h. Streptavidin–phycoerythrin (SA–PE) was then added to bind to the biotinylated detection antibodies on the bead surface. Afterwards, the plate was incubated for 30 min at room temperature, then washed three more times. The beads were resuspended in 100 µL of assay buffer, and the plate was shaken for 30 s. The microplate was immediately analyzed using a Bio-Plex 200 System (Bio-Rad Laboratories, Inc.; Hercules, CA, USA) equipped with Bio-Plex Manager Software 6.1. Apolipoprotein concentrations were calculated according to the appropriate standard curves and expressed in ng/mL.

### 2.4. Statistics

**Figure 2:**
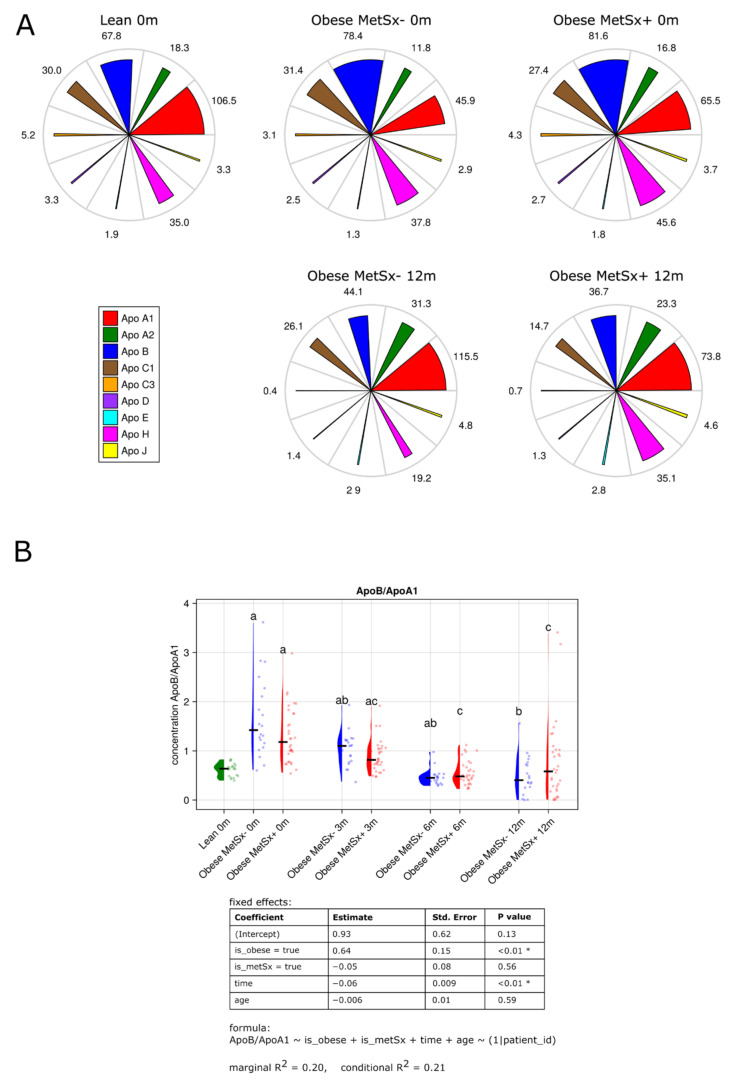
(**A**) Apolipoprotein distribution in blood plasma of patients. The radii are proportional to the median apolipoprotein concentration in a group (depicted as numbers around the circles and horizontal bars in Figure 3, Figure 4 and Figure 5). (**B**) ApoB/ApoA1 ratio in blood plasma of the patients. Lean subjects (*n* = 20); Obese MetSx-—obese patients without metabolic syndrome (*n* = 20); Obese MetSx+—obese patients with the accompanying metabolic syndrome (*n* = 32); 0 m—0 months (onset of the experiment); 12 m—12 months after the onset of the experiment. Significance markers (*p* < 0.05): a—vs. Lean (onset); b—vs. Obese MetSx- (onset); c—vs. Obese MetSx+ (onset); *—statistically significant coefficient (*p* < 0.05).

**Figure 3:**
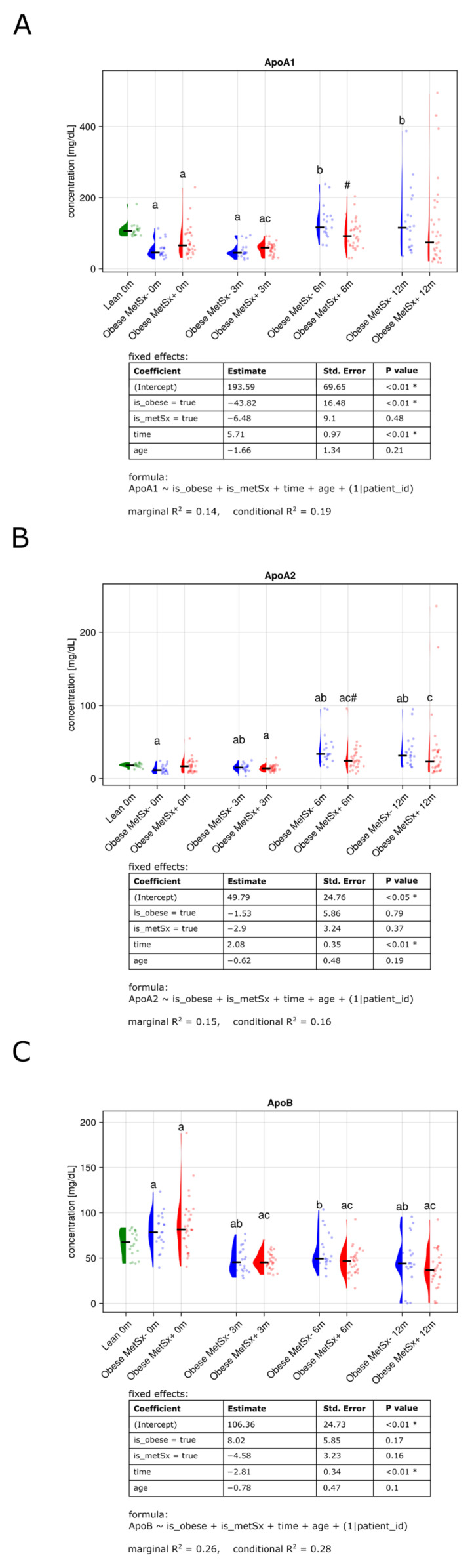
Apolipoprotein concentrations in blood plasma of patients. (**A**) Apolipoprotein A1; (**B**) apolipoprotein A2; (**C**) apolipoprotein B. Lean subjects (*n* = 20); Obese MetSx-—obese patients without metabolic syndrome (*n* = 20); Obese MetSx+—obese patients with the accompanying metabolic syndrome (*n* = 32); 0 m—0 months (onset of the experiment); 3, 6, and 12 m—3, 6, and 12 months after the onset of the experiment; a—difference vs. Lean at 0 m (*p* < 0.05); b—difference vs. Obese MetSx- at 0 m (*p* < 0.05); c—difference vs. Obese MetSx+ at 0 m (*p* < 0.05); #—difference of Obese MetSx- vs. Obese MetSx+ within a time point. *—statistically significant coefficient (*p* < 0.05).

**Figure 4:**
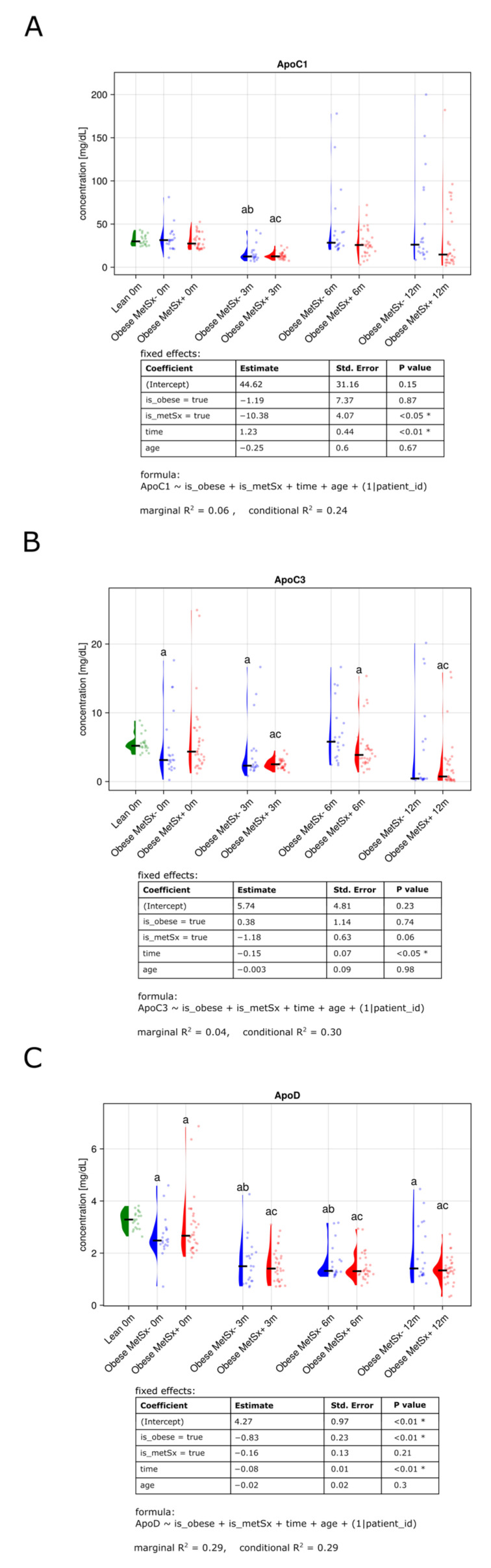
Apolipoprotein concentrations in blood plasma of patients. (**A**) Apolipoprotein C1; (**B**) apolipoprotein C3; (**C**) apolipoprotein D. Lean subjects (*n* = 20); Obese MetSx-—obese patients without metabolic syndrome (*n* = 20); Obese MetSx+—obese patients with accompanying metabolic syndrome (*n* = 32); 0 m—0 months (onset of the experiment); 3, 6, and 12 m—3, 6, and 12 months after the onset of the experiment; a—difference vs. Lean at 0 m (*p* < 0.05); b—difference vs. Obese MetSx- at 0 m (*p* < 0.05); c—difference vs. Obese MetSx+ at 0 m (*p* < 0.05); *—statistically significant coefficient (*p* < 0.05).

**Figure 5:**
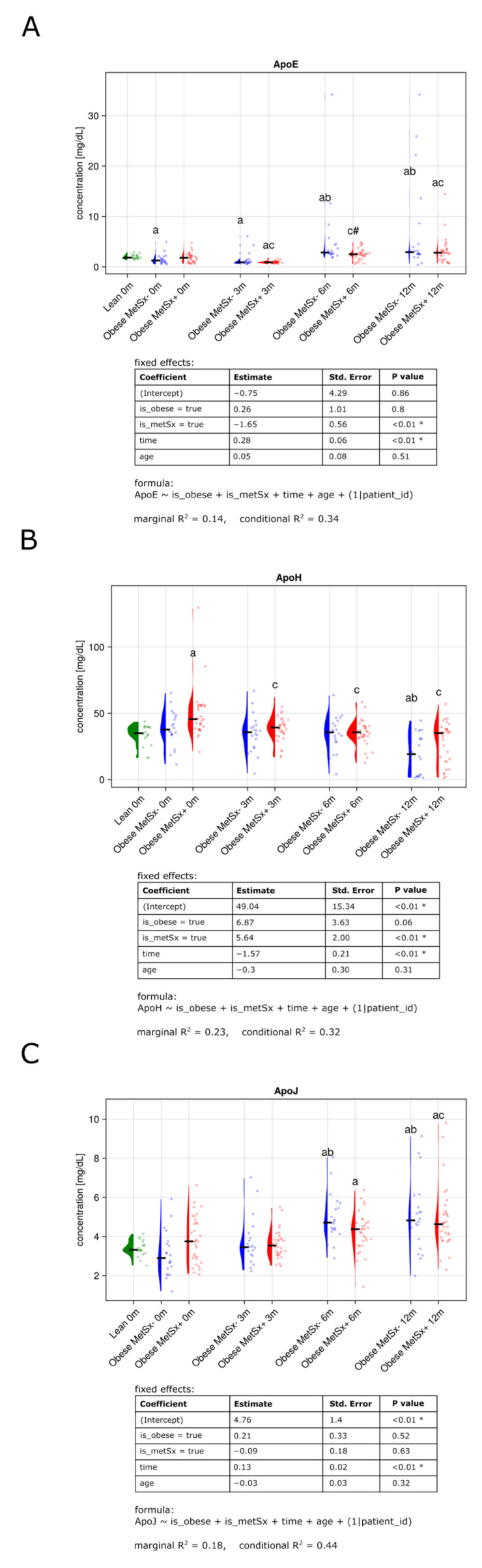
Apolipoprotein concentrations in blood plasma of patients. (**A**) Apolipoprotein E; (**B**) apolipoprotein H; (**C**) apolipoprotein J. Lean subjects (*n* = 20); Obese MetSx-—obese patients without metabolic syndrome (*n* = 20); Obese MetSx+—obese patients with accompanying metabolic syndrome (*n* = 32); 0 m—0 months (onset of the experiment); 3, 6, and 12 m—3, 6, and 12 months after the onset of the experiment; a—difference vs. Lean at 0 m (*p* < 0.05); b—difference vs. Obese MetSx- at 0 m (*p* < 0.05); c—difference vs. Obese MetSx+ at 0 m (*p* < 0.05); #—difference of Obese MetSx- vs. Obese MetSx+ within a time point. *—statistically significant coefficient (*p* < 0.05).

**Figure 6:**
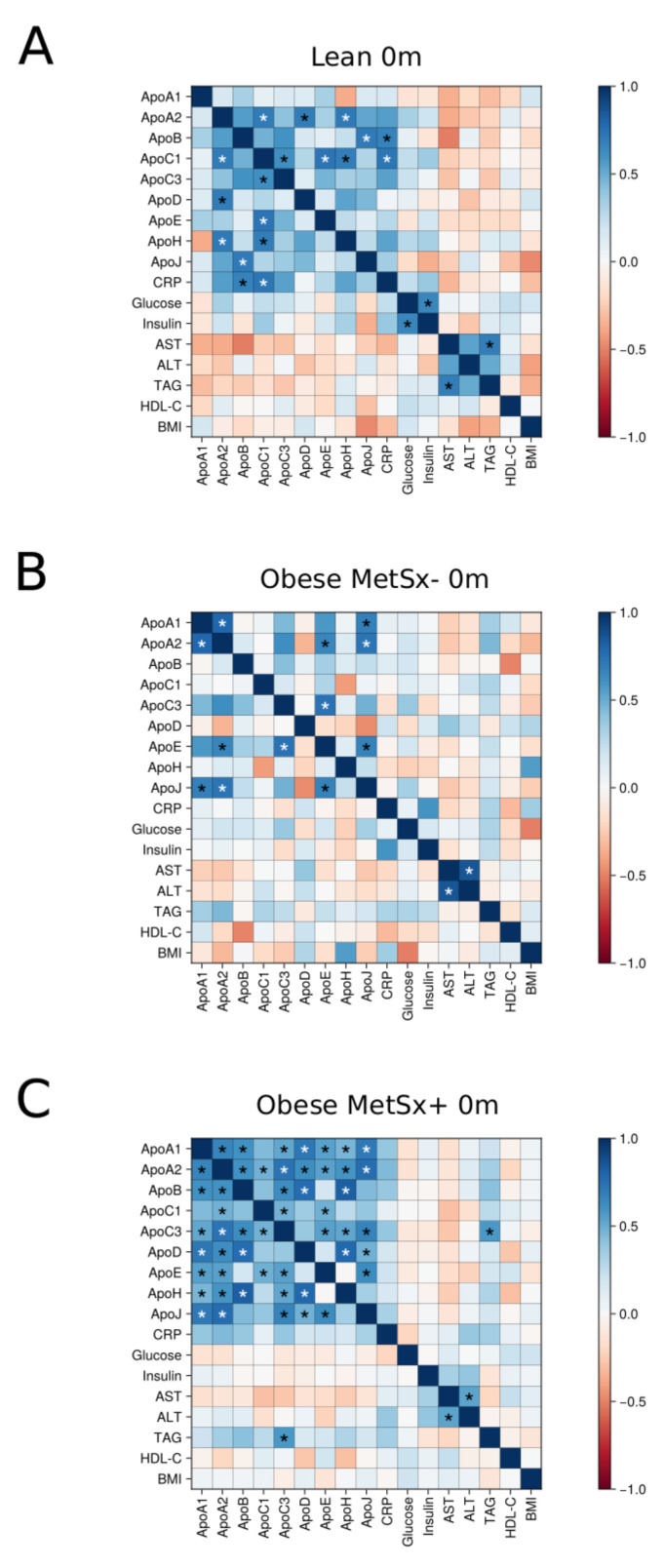
Pearson correlation coefficients presented in the form of a heatmap. Blue shading—positive correlation; red shading—negative correlation. (**A**) Lean at 0 m; (**B**) Obese MetSx- at 0 m; (**C**) Obese MetSx+ at 0 m. Lean subjects (*n* = 20); Obese MetSx-—obese patients without metabolic syndrome (*n* = 20); Obese MetSx+—obese patients with accompanying metabolic syndrome (*n* = 32); 0 m—0 months (onset of the experiment); *—correlation statistically significant (*p* < 0.05).

**Figure 7:**
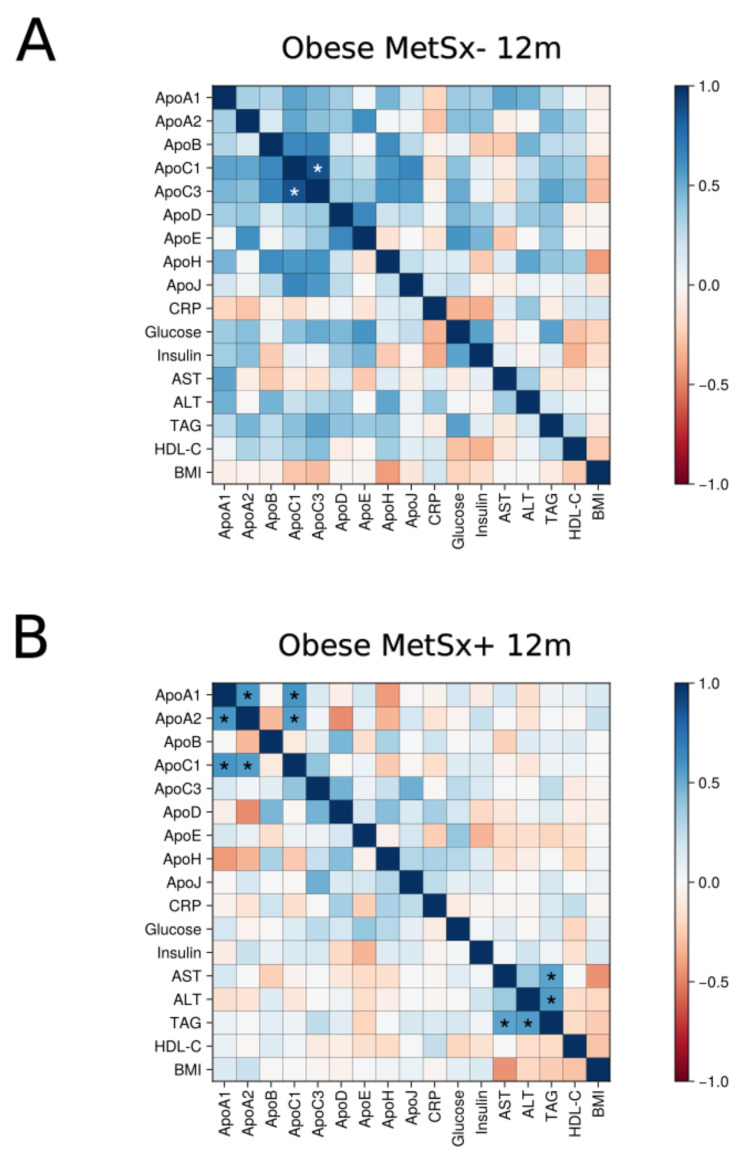
Pearson correlation coefficients presented in the form of a heatmap. Blue shading—positive correlation; red shading—negative correlation. (**A**) Obese MetSx- at 12 m; (**B**) Obese MetSx+ at 12 m (*n* = 20); Obese MetSx-—obese patients without metabolic syndrome (*n* = 20); Obese MetSx+—obese patients with accompanying metabolic syndrome (*n* = 32); 12 m—12 months after the onset of the experiment; *—correlation statistically significant (*p* < 0.05).

The data in Table 1 are presented as the mean and standard deviation. The data shown in Figure 2 are depicted in pie charts with radii proportional to the median apolipoprotein level in a group. Figure 3, Figure 4 and Figure 5 depict lipoprotein concentrations presented as a combination of a density plot (left side) and a scatter plot (right side). Figure 6 and Figure 7 represent Pearson’s correlation coefficients depicted as shades of blue (positive correlation) and red (negative correlation). Sample-size estimation was performed at the onset of the investigation based on the available literature data and expected effect sizes to be detected. Type I error was set at α ≤ 0.05, and power was set to be ≥0.8.

At the initial step of our analysis, a linear mixed-effects model was conducted with an apolipoprotein as an outcome variable. The explanatory variables (fixed effects) were: obesity, metabolic syndrome, and time (the three factors of interest), as well as the age of participants (a potential confounder). The analysis is summarized by tables placed in Figure 2, Figure 3, Figure 4 and Figure 5. For between-group comparison, a one-way ANOVA was conducted, followed by post hoc tests. The between-group comparisons for continuous variables were conducted with Student’s *t*-test or Wilcoxon test. The selection of the method was based on the normality (estimated with Shapiro–Wilk’s test) and variance homogeneity (checked by Fligner–Killeen test) criteria. To examine differences within the same group at different time points, a paired version of the test was applied. The correlation analysis (heatmaps) was conducted using Pearson’s correlation coefficients. *p*-values obtained from the above-mentioned methods were adjusted for multiple comparisons (Benjamini–Hochberg correction). A *p*-value ≤ 0.05 was considered to reach the level of statistical significance.

## 3. Results

### 3.1. Patients’ General Characteristics

Table 1 presents the basic data of the studied groups with respect to anthropometric measurements and standard blood-test results. The participants who met the diagnostic criteria for obesity were categorized into the ‘obese without metabolic syndrome’ group (designation of the group: Obese MetSx-, *n* = 20) and ‘obese with metabolic syndrome’ group (designation of the group: Obese MetSx+, *n* = 32). The two groups were characterized at the onset of the study and after sleeve gastrectomy (3, 6, and 12 months). Unsurprisingly, at time point 0, the obese groups had not only the previously mentioned greater BMI but also increased blood glucose (+30% and +49% for Obese MetSx- and Obese MetSx+ vs. Lean, respectively, *p* < 0.05), TAG (+24% for Obese MetSx+ vs. Lean, *p* < 0.05) and insulin (+2.88 fold and +3.18 fold for Obese MetSx- and Obese MetSx+ vs. Lean, respectively, *p* < 0.05) concentrations. Moreover, their CRP (+7.3 fold and +8 fold for Obese MetSx- and Obese MetSx+ vs. Lean, respectively, *p* < 0.05) and WBC (+24% for Obese MetSx+ vs. Lean, *p* < 0.05) levels were greater than in the lean patients. Aside from the above, the patients displayed greater waist (+79% and +82% for Obese MetSx- and Obese MetSx+ vs. Lean, respectively, *p* < 0.05) and hip (+49% and +51% for Obese MetSx- and Obese MetSx+ vs. Lean, respectively, *p* < 0.05) circumference, as well as reduced HDL-C content (−12% and −24% for Obese MetSx- and Obese MetSx+ vs. Lean, respectively, *p* < 0.05). Overall, the discussed parameters began to change 3 months after the bariatric surgery and approached the reference range 12 months after the intervention (Table 1).

### 3.2. Apolipoproteins

The conducted analysis (see tables in Figure 2, Figure 3, Figure 4 and Figure 5) revealed time as the most important factor influencing apolipoprotein levels, followed by metabolic syndrome and obesity.

In the first step of our study, we examined the overall composition of the examined apolipoproteins. In the lean subjects, they were arranged in descending order as follows: ApoA1, ApoB, ApoH, ApoC1, ApoA2, ApoC3, ApoJ, ApoD, and ApoE (Figure 2). In general, the apolipoprotein distribution also held in the intervention groups. However, there was one clear exception. Obese subjects had a greater share of ApoB at the expense of ApoA1 (Figure 2). The above was reversed (greater ApoA1 and smaller ApoB quota) twelve months after the therapeutic intervention (Figure 2).

The conducted analysis revealed lower ApoA1 concentrations in the obese groups at the onset of the experiment when compared to the lean subjects (−49% and −29% for Obese MetSx- and Obese MetSx+ vs. Lean at 0 m, respectively, *p* < 0.05, Figure 3A). The levels returned to the reference values roughly 6 months after the intervention. On the other hand, ApoA2 blood content displayed a different pattern, with approximately equal initial concentrations between the obese and lean subjects and an upsurge (+50–119%, on average, when compared with the lean group; Figure 3B) that was observed 6 and 12 months after the intervention. However, another data trend was observed for ApoB (Figure 3C): initially, both the obese groups displayed an increased level of the protein (+19% and +30% for Obese MetSx- and Obese MetSx+ vs. Lean, respectively, *p* < 0.05). Three months post bariatric surgery, its amount began to drop. Finally, it lowered by as much as 41% and 66% (for Obese MetSx- at 12 m vs. Obese MetSx- at 0 m; and Obese MetSx+ at 12 m vs. Obese MetSx+ at 0 m, *p* < 0.05) a year after the procedure, stimulating weight loss (Figure 3C).

The blood ApoC1 concentration was relatively stable, with a decrease 3 months after bariatric surgery (−51% and −66%, on average, for the Obese MetSx- 3 m and Obese MetSx+ 3 m groups, *p* < 0.05, Figure 4A). Unfortunately, it is not quite possible to say whether the unusual pattern is an assay artifact, a result of dietary changes, or a true biological phenomenon. Nevertheless, at the 6- and 12-month time points, the concentrations returned to normal (Figure 4A). On the other hand, the ApoC3 concentration appeared to be lower in the obese groups. In the case of Obese MetSx-, this was already evident at baseline (for Obese MetSx- vs. Lean, *p* < 0.05) and even intensified at the last time point (−23% and −53% for Obese MetSx- at 12 m and Obese MetSx+ at 12 m vs. Lean at 0 m, respectively, *p* < 0.05, Figure 4B). ApoD also showed this trend. Namely, we observed a slightly lower concentration at baseline, which seemed to decrease even more over time (−49% and −61% for Obese MetSx- 12 m and Obese MetSx+ 12 m vs. Lean, respectively, *p* < 0.05, Figure 4C).

Analysis showed that blood ApoE levels were lower in the obese groups compared with the lean group (Figure 5A). This was true at the first two time points (baseline and 3 months after surgery), particularly among obese individuals without metabolic syndrome (on average, −20% for Obese (-) at 0 m vs. Lean 0 m at 0 m, *p* < 0.05). Interestingly, this trend seemed to reverse, as 12 months after the intervention, we observed higher ApoE levels in both the obese groups when compared with the lean group or the same group at baseline (+68–350%, on average, with the median and interquartile range (IQR) for Lean at 0 m, Obese (-) at 12 m, and Obese (+) at 12 m being 1.9 (1.7–2.3), 2.9 (2.5–5.6) and 2.8 (1.5–3.4), respectively, Figure 5A). Furthermore, ApoH content (Figure 5B) was slightly higher in obese patients with metabolic syndrome at baseline (+46% for Obese MetSx+ at 0 m vs. Lean at 0 m, *p* < 0.05), followed by a decrease at later time points (−43% and −25% for Obese MetSx- at 12 m and Obese MetSx+ at 12 m vs. Lean, respectively, *p* < 0.05). The level of ApoJ was initially relatively stable in all the analyzed groups. The protein level increased at the 6- and 12-month follow-up points in both the obese groups (+47% and +44% for Obese MetSx- at 12 m vs. Lean 0 m and Obese MetSx+ at 12 m vs. Lean 0 m, respectively, *p* < 0.05).

### 3.3. Correlation Analysis

In addition to the above, we investigated possible inter-relationships between the analyzed variables (Table 2 and Figure 6 and Figure 7).

In the lean group (Figure 6A), we found statistically significant (*p* < 0.05) correlations between the following apolipoproteins: ApoC1 vs. ApoE, ApoC1 vs. CRP, ApoA2 vs. ApoH, ApoA2 vs. ApoC1, ApoB vs. ApoJ, ApoA2 vs. ApoD, ApoC1 vs. ApoH, and ApoC1 vs. ApoC3. The apolipoprotein concentrations were also correlated with some other variables—namely, ApoB vs. CRP (Figure 6A). Additionally, we found a relationship between blood glucose and insulin, as well as between AST and triacylglycerol content (Figure 6A).

In the Obese MetSx- group (Figure 6 and Figure 7), we observed a bunch of statistically significant (*p* < 0.05) correlations. For time 0, those were: AST vs. ALT, ApoA1 vs. ApoA2, ApoC3 vs. ApoE, ApoA2 vs. ApoJ, ApoA1 vs. ApoJ, ApoE vs. ApoJ, and ApoA2 vs. ApoE (Figure 6B). A significant correlation was observed only for ApoC1 vs. ApoC3 when measured 12 months after surgery (Figure 7A).

In the case of obese patients with metabolic syndrome (Obese MetSx+), there were clearly more statistically significant correlations (*p* < 0.05) at the onset of the experiment (*n* = 31, Figure 6C) than at its end (*n* = 6, Figure 7B). Those included (for 0 m): ApoB vs. ApoH, ApoD vs. ApoH, ApoA2 vs. ApoJ, ApoB vs. ApoD, ApoA2 vs. ApoC3, ApoA1 vs. ApoJ, ApoA1 vs. ApoD, ApoC3 vs. ApoJ, ApoA1 vs. ApoA2, ApoB vs. ApoC3, ApoE vs. ApoJ, ApoA2 vs. ApoD, ApoA2 vs. ApoB, ApoA1 vs. ApoB, ApoA2 vs. ApoH, ApoC3 vs. TAG, ApoA1 vs. ApoE, ApoA2 vs. ApoE, ApoC3 vs. ApoE, AST vs. ALT, ApoC3 vs. ApoH, ApoA1 vs. ApoC3, ApoC1 vs. ApoE, ApoD vs. ApoJ, ApoC1 vs. ApoC3, ApoA2 vs. ApoC1, and ApoA1 vs. ApoH (Figure 6C).

At the end of our experiment (12 months), five of the correlations were statistically significant (*p* < 0.05): ApoA1 vs. ApoC1, ApoA1 vs. ApoA2, ALT vs. TAG, ApoA2 vs. ApoC1, and AST vs. TAG (Figure 7B).

## 4. Discussion

In the first step of our study, we examined the appropriateness of the applied model. As expected, the patients from the intervention groups were characterized not only by excessive adiposity (BMI was roughly 45 kg/m^2^, compared to ≤25 kg/m^2^ in the reference group) but also by its metabolic consequences. These were clearly visible in the analyzed blood parameters, including high levels of fasting glucose, insulin, triacylglycerol, and HOMA-IR, alongside lowered HDL-C levels (Table 1). The above-mentioned variables returned fairly close to the reference range twelve months after the intervention (Table 1). It is worth noting that at the onset of our experiment, we observed a positive correlation (r = 0.66, *p* < 0.05) between blood glucose and insulin levels in lean patients but not in obese patients (Figure 6). Such an inter-relationship is to be expected in healthy individuals, as glucose is the main factor that triggers insulin release from pancreatic β-cells [9]. The hormone, in turn, facilitates the postprandial uptake of carbohydrates from the bloodstream into skeletal muscle and fat tissue [9]. The lack of such a connection between these two factors (insulin–glucose) may indicate reduced insulin sensitivity found in the tissues of obese subjects. Importantly, the correlation was also absent at the twelve-month checkup point (Figure 7), which suggests that, despite significant reduction in body mass, the underlying metabolic condition might not have been completely reversed.

The primary objective of this study was to evaluate changes in plasma apolipoprotein levels and related metabolic parameters in obese patients (with and without metabolic syndrome) in response to bariatric surgery. We noticed several interesting trends over the subsequent year after the therapeutic intervention. First, we observed different apolipoprotein distributions between the subjects at time 0 (Figure 2). The four most abundant apolipoprotein types were the same in each group: ApoA1, ApoB, ApoH, and ApoC1. However, the first two displayed an interesting pattern. The dominant apolipoprotein in the lean group was ApoA1. This contrasts with the obese groups, which had a significantly higher proportion of ApoB (Figure 2). It is worth noting that twelve months after the bariatric surgery, a shift towards higher ApoA1 expression was observed in obese patients. The above appears to be of paramount importance because apolipoprotein A1 is a main component of HDL-C (‘good cholesterol’), known for its role in reverse cholesterol transport and LCAT activation [10]. On the other hand, apolipoprotein B is a key component of LDL-C particles (‘bad cholesterol’) and chylomicrons [11]. This suggests that lipid homeostasis was disturbed in the obese patients at the onset of the experiment and restored following the therapeutic intervention. The above is also bolstered by the data presented in Figure 3A (ApoA1) and 3C (ApoB), as well as in Table 1 (HDL-C and TAG). Based on these parameters, we postulate that the improvement in lipid metabolism occurred around 6 months post intervention. The postulated phenomenon is also well supported by the available literature [12,13,14], like the INTERHEART study [14] published in *The Lancet*. The research is a large-scale case–control study conducted in 52 countries. It was designed to examine the main risk factors for myocardial infarction (MI). The investigation found that the ApoB/ApoA1 ratio is a powerful predictor of acute MI. Interestingly, the odds ratio (OR) for ApoB/ApoA1 was greater (OR = 3.25) than for the other examined ‘classic’ hazards, like cigarette smoking (OR = 2.87), diabetes (OR = 2.37), hypertension (OR = 1.91), and abdominal adiposity (OR = 1.12) [14]. Furthermore, a study by Walldius and Jungner [12] confirmed these findings. The authors juxtaposed the results of several dozen scientific papers (including the aforementioned INTERHEART study) and concluded that the ApoB/ApoA1 ratio is a useful marker not only for assessing the risk of MI (both fatal and non-fatal) but also of stroke, atherosclerosis, heart failure, aneurysm, and renal failure [12]. This index better represents the balance between atherogenic and antiatherogenic lipoprotein particles than the conventionally used HDL and LDL cholesterol concentrations [12].

Interestingly, not all apolipoproteins differed in levels between obese and lean patients at baseline. This included ApoA2 (Figure 3B), ApoE, and ApoJ (Figure 5). Initially, the concentrations of these proteins were similar to those in the lean group, which changed over time, as their levels began to increase approximately 6 months after the surgery (Figure 2 and Figure 5).

Apolipoprotein A2 (ApoA2) is an abundant apolipoprotein in HDL-C particles, second only to ApoA1. Its role in the development of different pathologies remains uncertain, as studies often provide ambiguous or contradictory data. On the one hand, a paper by Zvintzou et al. showed that transient overexpression of ApoA2 in C57BL/6 mice resulted in pleiotropic effects on lipid and glucose metabolism, most of which were considered beneficial [15]. The authors found an increased rate of oxidative phosphorylation processes in WAT (white adipose tissue), together with a higher HDL antioxidant capacity and improved intraperitoneal glucose tolerance [15]. However, the above findings stand in contrast to some other research. For instance, Castellani and co-workers demonstrated that transgenic mice overexpressing ApoA2 had approximately two-fold greater fasting plasma insulin levels and reduced 2-deoxyglucose uptake in their skeletal muscles—clear signs of insulin resistance [16]. Moreover, other researchers observed that mice had pronounced atherosclerotic lesions, despite elevated blood HDL-C levels, which are conventionally considered to act protectively against the condition [17]. The above could be explained by the fact that HDL particles are made of either ApoA1 alone or a mixture of both apolipoproteins [18,19]. The composition is an important factor that determines the biological properties of HDL. The presence of ApoA1, itself, results in larger HDL particles and improved efficiency in reverse cholesterol transport [19], resulting in HDL’s superior protective properties against atherosclerosis. On the other hand, the combination of the two apolipoproteins (ApoA1 and ApoA2) results in smaller HDL particles that tend to become dysfunctional in the inflammatory conditions accompanying chronic obstructive pulmonary disease [19] and possibly also obesity (higher CRP concentration in our study). For these reasons, most studies focus on ApoA1 (and the aforementioned ApoB/ApoA1 ratio) and its role in adiposities.

ApoE and ApoJ exhibit a pattern similar to that of ApoA2, i.e., roughly at the reference level at the onset, with a clear increase six and twelve months after the therapeutic intervention. Apolipoprotein E is a multifunctional protein best known for its role in the clearance of TAGs from circulation and promotion of their accumulation in adipose tissue [20]. In line with that notion, Hofmann et al. demonstrated that C57BL/6 apoE+/+ mice fed a chow diet were characterized by lower fasting blood TAG and cholesterol concentrations when compared with their ApoE-/- littermates [21]. Moreover, the animals displayed a greater percentage of body fat despite a roughly equal body weight when contrasted with ApoE-deficient rodents [21]. Together with our results (Figure 5A), the above suggests accelerated clearance of lipid moieties from the bloodstream and an overall reduction in cardiovascular risk for patients after bariatric surgery. ApoJ is produced mostly in the liver and is found in both HDL and LDL particles [22]. Although its function in lipoprotein metabolism has remained elusive in recent years, it has attracted attention as an important regulator of skeletal-muscle insulin sensitivity. For instance, Seo et al. generated knock-out mice with liver-specific ApoJ ablation (L-ApoJ-/-) [22]. As expected, the animals were characterized by reduced circulating ApoJ content but without changes in the liver or blood TAG and cholesterol volumes. Despite the lack of severe lipid changes, these rodents developed peripheral insulin resistance, particularly in skeletal muscle. The condition was established based on increased fasting levels of plasma glucose (despite the unchanged concentration of insulin), as well as a weaker response in the insulin-tolerance test [22]. Interestingly, insulin resistance was independent of adiposity (no difference in body weight or fat mass between L-ApoJ-/- and the controls) and affected all the examined tissues (reduced phosphorylation of IRS in the liver, skeletal muscle, and fat depots) [22]. Such an increase in ApoJ concentration could be an indicator of the improved insulin sensitivity observed in our subjects a few months after the therapeutic intervention (Table 1). However, this needs to be confirmed by further studies, as not all the available research is in line with that notion. Rodrigues and co-workers [23] did not detect such a significant change in ApoJ concentration 12 months after RYGB surgery. The discrepancy in the findings could be attributed to small sample size, as the study included only seven subjects [23].

Yet another pattern was observed for ApoC3 and ApoD (Figure 4B,C). Their concentrations were initially slightly below control levels and continued to drop throughout the experiment. This downward trend was observed at three months and reached its lowest value at the final time point. The above is of importance, since recent investigations indicate that ApoC3 plays a key role in the regulation of the body’s triacylglycerol levels, governed largely by its inhibitory action on lipoprotein lipase (LPL). LPL is an enzyme responsible for breaking down TAGs [24]. High ApoC3 expression inhibits this action, reduces the clearance of lipids from the bloodstream, and leads to hypertriglyceridemia [24]. Additionally, ApoC3 may serve as a biomarker of cardiovascular diseases, as shown in studies on transgenic animals. For instance, Zha and colleagues [25] generated ApoC3 knockout rabbits using CRISPR gene editing. It is worth noting that when kept on a standard chow diet, the animals had a ~50% lower triacylglycerol concentration than their wild-type counterparts [25]. TAGs remained lower (by roughly 70%), even when the animals were placed on a high-fat diet for 6 and 12 weeks. The rabbits were also protected from atherosclerosis, as evidenced by lower aortic tree lesion coverage, reduced intima thickening, or reduced macrophage accumulation [25]. Apolipoprotein D is also an important biologically active molecule with potential involvement in many diseases, like cancer [26] and hypothyroidism [27]. However, its role in obesity is less well known, with studies in animals providing contradictory findings [28,29]. Still, the data on apolipoprotein D and its impact on cardiovascular diseases seem to be more conclusive. Annema et al. measured ApoD levels in a cohort of over five hundred Caucasians, with a follow-up period of almost six years, and found them to be prognostic of coronary artery disease (CAD). The researchers compared patients in the highest quartile (Q3) of baseline ApoD with those in the lowest quartile (Q1) and found that the former group had significantly elevated risks of adverse cardiovascular events (HR = 2.52) and cardiovascular mortality (HR = 5.47) [30]. The above is in line with other studies that have reported greater deposition of ApoD in the atherosclerotic lesions of people with CAD, as well as in mouse models of the condition [31].

Interestingly, apolipoprotein H concentrations also displayed changes that somewhat resembled the ones reported for ApoC3 and ApoD. Initially, ApoH content was slightly greater in the obese groups than in the lean subjects. However, the above reached the level of statistical significance only in the patients with metabolic syndrome (Figure 5B). Over time, the amount of apolipoprotein began to drop (in Obese MetSx+ patients, as early as 3 months after the surgery, whereas in Obese MetSx- patients, it was observed only at month 12). It is worth noting that apolipoprotein H is a protein whose exact biological function still needs to be elucidated. As regards obesity and its comorbidity, Castro et al. demonstrated [32] that its blood level was elevated in a cohort of non-smoking type 2 diabetic subjects with metabolic syndrome when compared with a healthy population. Moreover, the authors found that ApoH concentration was significantly associated with the amounts of blood triacylglycerols (r = 0.337, *p* < 0.001) and VLDL-triacylglycerols (r = 0.359, *p* < 0.001). Other findings also seem to support the role of ApoH in lipid disturbances. Liu and co-workers reported that apolipoprotein H-deficient mice (ApoH-/-) displayed spontaneous steatohepatitis (as evidenced by histopathological analysis and Oil Red O staining), which was further worsened by ethanol consumption [33]. Others point out that oxidized LDL/β2-blycoprotein 1 complexes (β2-blycoprotein 1 is another literature name for Apo H) are the principal lipoproteins present in the atherosclerotic lesions of patients with coronary artery disease [34,35]. Although further studies on the role of ApoH are still needed, the above information is consistent with our results (Figure 5B). Moreover, it indicates improvements in the lipid profiles and cardiovascular fitness of the patients from Obese MetSx+ group in response to the therapeutic intervention.

## 5. Conclusions

In summary, we believe our study is the first to precisely characterize the apolipoprotein profile of patients after bariatric surgery. Importantly, the study was conducted in three metabolically separate groups (lean patients and obese patients without and with metabolic syndrome) and at four distinct time points (0, 3, 6, and 12 months post therapeutic intervention). We observed a few interesting patterns in the data. Most notably, obese patients had a significantly higher ApoB/ApoA1 ratio at baseline, which returned to the reference level over the following twelve months. The above is of great clinical importance, as an elevated ratio is believed to be an important predictor of adverse cardiovascular events, such as atherosclerosis and myocardial infarction, which are particularly common in individuals with obesity. Interestingly, the plasma concentrations of many investigated apolipoproteins appeared to be relatively stable at the onset of the experiment, with subsequent changes observed at later time points. In line with that notion, we detected statistically significant drops in the levels of ApoC3, ApoD, and ApoH, with shifts that occurred as early as three months after the therapeutic intervention. All these proteins have roles in the development and progression of vascular pathologies like atherosclerosis and coronary artery disease, as documented in the literature. Hence, reductions in their volumes may be interpreted as a sign of improved cardiovascular fitness of our patients. On the other hand, the levels of ApoA2, ApoE, and ApoJ increased with over time course of our study.

### Limitations

Our study is not without certain limitations. First, the dataset does not contain information on the patients’ physical activity status. Based on general epidemiological data, we expect that physical activity was lower in the obese groups compared to the lean patients. Moreover, after the surgery, the patients were advised to be physically active (~150 min of moderate physical activity per week); however, activity was not recorded in this study. We managed to obtain blood samples from lean patients only for the initial time point (0 months), without the subsequent follow-up (3, 6, and 12 months), which could be of additional value. We believe that future studies would benefit from a longer time span (up to 24 months) and a greater number of patients per group. Lastly, other potential limitations of our study include the single-center design, the lack of direct cardiovascular outcomes, and the lack of recording of patient diets.

## Figures and Tables

**Table 1:** The main clinical characteristics of the patients. Data are presented as mean ± SD. Lean (*n* = 20); Obese MetSx-—obese without metabolic syndrome (*n* = 20); Obese MetSx+—obese with metabolic syndrome (*n* = 32). Significance markers (*p* < 0.05): a—vs. Lean (onset); b—vs. Obese MetSx- (onset); c—vs. Obese MetSx+ (onset), #—Obese MetSx- vs. Obese MetSx+ (within a given time point). ALT—alanine aminotransferase; AST—aspartate transaminase; BMI—body mass index; CRP—C-reactive protein; DP—diastolic pressure; HDL-C—high-density lipoprotein; HGB—hemoglobin; PLT—platelet count; RBC—red blood cell count; SP—systolic pressure; TAG—triacylglycerol; WBC—white blood cell count.

	Lean	Obese (-)	Obese (+)	Obese (-)	Obese (+)	Obese (-)	Obese (+)	Obese (-)	Obese (+)
	Onset			3 Months After		6 Months After		12 Months After	
Age [yrs]	50.9 ± 3.2	52.8 ± 2.6	51.1 ± 3.4	---	---	---	---	---	---
Weight [kg]	67.6 ± 6.3	122.7 ± 17.0 a	121.4 ± 15.1 a	100.2 ± 11.5 ab	99.4 ± 12.9 ac	88.4 ± 10.5 ab	88.9 ± 11.1 ac	79.0 ± 9.7 ab	79.5 ± 10.7 ac
BMI [kg/m^2^]	23.6 ± 1.2	46.5 ± 5.4 a	45.2 ± 5.2 a	38.0 ± 3.4 ab	37.0 ± 4.4 ac	33.5 ± 3.1 ab	33.1 ± 3.9 ac	29.9 ± 3.0 ab	29.7 ± 4.0 ac
Waist [cm]	74.2 ± 4.5	133.0 ± 8.5 a	135.7 ± 9.6 a	113.5 ± 7.3 ab	118.1 ± 9.2 ac	101.0 ± 7.5 ab	107.4 ± 9.0 ac#	90.8 ± 7.5 ab	95.4 ± 8.1 ac
Hip [cm]	93.0 ± 6.2	138.6 ± 5.7 a	140.8 ± 11.1 a	121.3 ± 4.9 ab	124.6 ± 9.8 ac	110.4 ± 6.4 ab	114.9 ± 8.2 ac	101.8 ± 6.6 ab	104.7 ± 7.8 ac
Glucose [mg/dL]	74.0 ± 6.1	96.0 ± 7.1 a	110.2 ± 19.7 a#	90.4 ± 6.8 ab	97.8 ± 14.8 ac#	87.0 ± 6.0 ab	95.2 ± 13.9 ac#	85.2 ± 6.6 ab	91.7 ± 6.9 ac#
Insulin [μU/mL]	5.3 ± 2.5	20.6 ± 6.7 a	22.2 ± 6.8 a	10.3 ± 3.5 ab	11.8 ± 6.0 ac	8.6 ± 2.1 ab	9.3 ± 3.2 ac	8.2 ± 2.5 ab	8.5 ± 3.2 ac
AST [U/L]	23.8 ± 4.4	29.2 ± 12.0	26.8 ± 9.2	24.2 ± 9.2	23.6 ± 7.5	20.6 ± 7.0 b	20.2 ± 8.5 ac	20.1 ± 7.9 ab	19.3 ± 7.1 ac
ALT [U/L]	21.6 ± 3.4	25.1 ± 9.6	23.7 ± 8.9	23.0 ± 7.1	22.1 ± 5.7	21.4 ± 8.1	22.2 ± 9.6	21.4 ± 5.7	21.1 ± 7.4
TAG [mg/dL]	135.4 ± 8.7	135.8 ± 14.2	171.0 ± 37.7 a#	129.5 ± 18.8	149.7 ± 23.7 ac#	123.5 ± 10.9 ab	136.5 ± 16.1 c#	123.0 ± 8.4 ab	128.5 ± 10.7 ac
HDL-C [mg/dL]	58.8 ± 5.1	52.0 ± 5.8 a	44.6 ± 7.6 a#	52.4 ± 4.1 a	46.4 ± 8.5 a#	52.8 ± 6.4 a	52.5 ± 10.8 ac	55.4 ± 5.0	54.4 ± 7.8 ac
WBC [10^3^/mm^3^]	7.4 ± 1.1	7.9 ± 1.2	9.2 ± 1.7 a#	6.8 ± 1.5 b	7.3 ± 1.7 c	6.2 ± 1.0 ab	7.3 ± 1.8 c#	6.0 ± 1.1 ab	6.7 ± 1.6 c
RBC [10^6^/mm^3^]	4.5 ± 0.3	4.7 ± 0.5	4.7 ± 0.5	4.7 ± 0.4	4.8 ± 0.4	4.8 ± 0.5	4.8 ± 0.4	4.7 ± 0.4	4.7 ± 0.5
HGB [g/dL]	13.9 ± 1.0	13.0 ± 1.1	13.4 ± 1.0	13.1 ± 1.3	13.2 ± 0.9	13.2 ± 1.5	13.5 ± 1.1	14.0 ± 1.1 b	13.6 ± 1.5
PLT [10^3^/mm^3^]	287.4 ± 16.8	270.0 ± 58.9	261.5 ± 62.5	257.9 ± 55.9	248.8 ± 51.9 a	282.0 ± 68.9	263.7 ± 61.2	235.8 ± 55.6 a	231.6 ± 55.3 a
CRP [mg/L]	1.8 ± 0.6	15.0 ± 12.2 a	16.3 ± 13.9 a	14.9 ± 14.8 a	19.8 ± 17.8 a	5.1 ± 5.5 b	9.1 ± 16.1 c	4.1 ± 5.9 b	9.3 ± 16.1 ac
SP [mmHg]	123.0 ± 5.7	123.8 ± 4.8	135.5 ± 10.3 a#	124.8 ± 7.7	130.9 ± 8.0 ac#	123.2 ± 5.2	129.5 ± 8.4 ac#	123.0 ± 4.7	128.1 ± 8.0 ac#
DP [mmHg]	79.8 ± 3.0	82.0 ± 4.1	90.9 ± 8.6 a#	82.0 ± 4.1	86.9 ± 7.8 ac#	80.8 ± 2.9	86.2 ± 5.7 ac#	80.2 ± 2.0	85.6 ± 5.4 ac#
HOMA-IR	1.0 ± 0.5	4.9 ± 1.7 a	6.0 ± 2.0 a#	2.3 ± 0.8 ab	2.9 ± 1.6 ac	1.9 ± 0.5 ab	2.2 ± 0.8 ac	1.7 ± 0.6 ab	1.9 ± 0.7 ac

**Table 2:** Correlation table. Only statistically significant correlations (* in Figure 5 and Figure 6) are included. BH—Benjamini–Hochberg multiplicity correction.

Correlation	Pearson’s r	Adjusted *p*-Value (BH Correction)
Lean at 0 m		
ApoC1 vs. ApoE	0.74	0.01
ApoC1 vs. CRP	0.73	0.01
ApoA2 vs. ApoH	0.72	0.01
ApoA2 vs. ApoC1	0.71	0.01
ApoB vs. ApoJ	0.71	0.01
ApoA2 vs. ApoD	0.7	0.01
AST vs. TAG	0.7	0.01
ApoC1 vs. ApoH	0.69	0.01
ApoB vs. CRP	0.68	0.02
Glucose vs. Insulin	0.66	0.02
ApoC1 vs. ApoC3	0.62	0.05
Obese MetSx- at 0 m		
AST vs. ALT	0.86	0.00
ApoA1 vs. ApoA2	0.79	0.00
ApoA2 vs. ApoJ	0.77	0.00
ApoC3 vs. ApoE	0.75	0.01
ApoA1 vs. ApoJ	0.71	0.01
ApoE vs. ApoJ	0.67	0.03
ApoA2 vs. ApoE	0.66	0.03
Obese MetSx+ at 0 m		
ApoB vs. ApoH	0.82	0.00
ApoD vs. ApoH	0.79	0.00
ApoA2 vs. ApoJ	0.77	0.00
ApoB vs. ApoD	0.77	0.00
ApoA2 vs. ApoC3	0.74	0.00
ApoA1 vs. ApoJ	0.72	0.00
ApoA1 vs. ApoD	0.72	0.00
ApoC3 vs. ApoJ	0.67	0.00
ApoA1 vs. ApoA2	0.66	0.00
ApoB vs. ApoC3	0.63	0.00
ApoE vs. ApoJ	0.63	0.00
ApoA2 vs. ApoD	0.62	0.00
ApoA2 vs. ApoB	0.61	0.00
ApoA1 vs. ApoB	0.61	0.00
ApoA2 vs. ApoH	0.6	0.00
ApoC3 vs. TAG	0.58	0.00
ApoA1 vs. ApoE	0.55	0.01
ApoA2 vs. ApoE	0.54	0.01
ApoC3 vs. ApoE	0.54	0.01
AST vs. ALT	0.53	0.01
ApoC3 vs. ApoH	0.52	0.01
ApoA1 vs. ApoC3	0.52	0.01
ApoC1 vs. ApoE	0.49	0.03
ApoD vs. ApoJ	0.48	0.03
ApoC1 vs. ApoC3	0.48	0.03
ApoA2 vs. ApoC1	0.48	0.03
ApoA1 vs. ApoH	0.47	0.04
Obese MetSx- at 12 m		
ApoC1 vs. ApoC3	0.89	0.00
Obese MetSx+ at 12 m		
ApoA1 vs. ApoC1	0.59	0.03
ApoA1 vs. ApoA2	0.59	0.03
ALT vs. TAG	0.56	0.03
ApoA2 vs. ApoC1	0.56	0.03
AST vs. TAG	0.54	0.04

## Data Availability

The original contributions presented in this study are included in the article. The raw data supporting the conclusions of this article will be made available by the authors upon reasonable request. Further inquiries can be directed to the corresponding author.
